# Editorial: Application and research progress of avian models in neuroscience

**DOI:** 10.3389/fnmol.2023.1319308

**Published:** 2023-10-24

**Authors:** Wei Meng

**Affiliations:** Jiangxi Key Laboratory of Organic Chemistry, Jiangxi Science and Technology Normal University, Nanchang, China

**Keywords:** bird, avian models, songbird, vocal learning, evolution, neural projection, estrogen, serotonergic system

Over recent decades, different types of animal models, from arthropods to primates, have been applied to neuroscience research, making irreplaceable contributions to reveal the neural basis of various behaviors. Aves are one of the most prosperous amniotes (totaling over 10,000 species) globally, which has evolved in parallel with mammals. Although avian and mammalian brains are constructed in very different ways, the sensory information processing, cognition, behavior, and related neural control pathways of some bird species are highly similar to those of mammals, including primates and even humans. For example, the brains of birds and mammals have conserved visual and auditory pathways (Ball and Balthazart, 2021); corvids and parrots have some advanced cognitive abilities that are highly comparable to primates (Clayton and Emery, 2015); songbirds have homologous vocal learning-related pathways to humans and song-learning behavior similar to human language learning (Davenport and Jarvis, 2023).

Decades of research have shown that birds can exhibit very complex cognitive abilities, including visual discrimination, orientation and navigation, food storage, vocal learning and communication, complex social interaction, and problem-solving abilities, some of which are highly similar to human behaviors that are unmatched by other animal models. The advantages of avian models not only can reveal the neural basis of behaviors more extensively but also provide valuable insights into our own behaviors than we thought. At the same time, it can greatly promote our understanding of the evolution of cognitive function. With the support of advanced techniques of electrophysiology, histology, pharmacology, optogenetics, molecular biology, and neuroimaging, significant progress has been made in revealing the neural basis of some avian behaviors. However, neurobiological data obtained from avian models are still limited, compared with other animal models, especially mammalian models. The lack of knowledge about the relevant neural pathway connectivity and genetic information in the brains of many birds also limits the application of avian models in neuroscience.

The special Research Topic “*Application and research progress of avian models in neuroscience*” of *Frontiers in Molecular Neuroscience* brings together three reviews from different perspectives and one original research to report new findings on the development of avian neurobiology.

Vocal production learning is a kind of information communication ability that only few animal species possess. It refers to the behavior of animal individuals forming new vocal signals or modifying existing vocal signals based on auditory feedback from other individuals' vocalizations and their own vocalizations. Besides humans, the most striking examples of vocal production learning are songbirds, parrots, and hummingbirds in birds, and cetaceans and bats in mammals (Beecher, 2021). In two strikingly similar examples, both human toddlers and songbird chicks need to memorize the conspecific vocal signals in the early stages of life, driven by a seemingly instinctive learning process, and undergo sensorimotor practice to match their own vocalizations with auditory memory through auditory feedback. However, there are still many unanswered questions about the evolution of vocal production learning. Many publications discussed the vocalization behavior of different species, compared the neural basis and physiological and genetic mechanisms of vocal learning behavior in a few species with vocal learning ability (including humans and songbirds), and proposed hypotheses of the evolution of vocal learning. From a novel and unique environmental and ecological perspective, Brenowitz and Beecher's new review discusses the potential costs of vocal production learning and their possible implications for the evolution of vocal production learning. They suggest that young songbirds and human brains may be able to selectively focus on conspecific vocalizations to reduce the risk of replication errors. In addition, many songbird species, like humans, have a sensitive period of vocal learning during their infancy, which is another way to reduce the costs of vocal production learning. Moreover, plasticity of forebrain structures related to vocal production learning in songbirds during the breeding season can reduce the energy cost of these brain regions. Brenowitz and Beecher further argue that in habitats where visual information transmission is limited, auditory information transmission becomes the primary means of long-distance communication between individuals, while the amount of information that innate non-learned vocalizations can carry is very limited. The evolution of vocal production learning can enable certain species to encode more complex and even infinitely variable acoustic signals, which reflects the relative costs and benefits of learning due to the limitations of ecological environmental factors on specific species. Under the pressure of ecological selection, species that have evolved in vocal production learning may include: (1) Species that communicate primarily or only through auditory signals over long distances (separating the sender and receiver). (2) Species that need to converge or diverge from other group members by modifying their vocal signals, such as dolphins, seals, bats, elephants. (3) Species that would benefit from increasing the complexity of vocal signals, such as songbirds and whales. The perspective of this article provides a new integrated framework for understanding the evolution of vocal production learning.

Next, the review presented by Zhang, Zhou et al. is also about animal vocal learning, but focuses more on the comparison of similarities between songbird singing and human speech. The article begins with an overview of the current understanding of mammalian and avian vocal learners, including newly discovered potential vocal learners. Based on existing research data, the following part of the paper focuses on the rare similarities in vocal learning between humans and songbirds, which are known to have different evolutionary origins. The song learning process of young songbirds is similar to the speech learning process of human infants, including the sensory stage and the sensorimotor stage, which require the participation of auditory feedback. Additionally, humans and some songbirds, such as zebra finches, have similar vocal learning critical periods and have greater learning abilities in infancy. Another important feature of human language is its complex grammatical structure. Some species of songbirds, such as Bengalese finches, can flexibly and purposefully change the order of syllables in their songs, just like humans control the order of words in language. Although there are insurmountable differences in the structure and cell types of the cerebral cortex between songbirds and humans, the coordination and interaction between the forebrain vocal motor pathway and the brainstem innate vocal pathway are indispensable for the production of autonomous vocalization, and the vocal learning pathway and the vocal motor pathway have similar neural connections, and both receive auditory information from the auditory system. This article further compares the neural basis of human speech and songbird singing, and in detail compared the similarities of projection connections, functions, neural activities, cell types, genome and transcriptome between each human speech-related brain region and songbird song-related neural nucleus in the brainstem innate vocal control pathway, forebrain vocal motor pathway and vocal learning pathway, including more recent findings. The article concludes with outlining, prospective topics of relevance for the future research.

Estrogen in the animal brain, mainly estradiol 17β-Estradiol (E2), is converted from androgens under the action of aromatase, and can affect brain function and behavior (Balthazart and Ball, 2006). In songbirds, E2 receptor ERα and G-protein-coupled membrane-bound estrogen receptor (GPER) are expressed in song control nuclei (Jacobs et al., 1999; Acharya and Veney, 2012), and E2 can affect singing behavior (Alward et al., 2016). The original research paper contributed by Zhang, Sun et al. to this Research Topic is the first evidence to prove that E2 can regulate the excitability of projection neurons in the song premotor nucleus robust nucleus of the arcopallium (RA) in adult songbirds. In this study, whole-cell patch-clamp recordings of adult male zebra finch RA projection neurons were performed and combined with pharmacological detection. The findings indicated that E2 has the ability to lower the spontaneous and evoked action potential firing rate of RA projection neurons, induce hyperpolarization of the resting membrane potential, and decrease the membrane input resistance. At the same time, it was found that GPER agonist G1 can mimic the action of E2 on RA projection neurons, while GPER antagonistist G15 can block the effect of E2, indicating that E2 reduces the excitability of RA projection neurons via GPER. These results contribute to understanding the regulation of E2 on the intrinsic membrane properties of songbird RA projection neurons and its receptor mechanisms.

Serotonin (5-hydroxytryptamine, 5-HT) is an evolutionarily conserved neurotransmitter present in the central nervous system of all vertebrates. It is now known that 5-HT is widely involved in the regulation of mammalian brain functions, including cognition, behavior, and emotion (Bacque-Cazenave et al., 2020). However, the knowledge of the central serotonergic system in non-mammalian vertebrates is still very limited. With chickens as the main bird model, the review of Fujita et al. summarizes our current understanding of the anatomical and molecular characteristics of the central serotonergic system in birds. They first outline our current knowledge of mammalian serotonergic systems, laying the groundwork for a comparative introduction of avian serotonergic systems in the following text. The next part discusses our current understanding and controversy on the homology of avian and mammalian brains, mainly focusing on the hippocampal formation and amygdala, two important structures associated with cognition, behavior, and emotion. Furthermore, based on the authors' recent research results on the molecular biology of serotonergic systems in the chicken brain, the distribution of serotonergic systems in the brains of birds and mammals is compared and analyzed. The potential interaction between serotonergic systems and dopaminergic systems in birds is also mentioned. Finally, the authors briefly discuss the function of serotonergic system in bird brain. In particular, the researches on the expression and function of related genes suggest that the behavior regulation of serotonergic systems in birds is similar to that in mammals. This review is of broad interest to the avian neurobiology community, and can provide comparison and reference for the study of mammalian central serotonergic system.

## Author contributions

WM: Funding acquisition, Project administration, Resources, Writing – original draft, Writing – review & editing.
